# Study on adolescents’ smoking intentions: the influence of peer smoking and the mediating roles of beliefs and attitudes toward smoking behavior

**DOI:** 10.3389/fpubh.2026.1775438

**Published:** 2026-03-02

**Authors:** Liangliang Gong, Zanzan Zhang, Liping Wang, Zhidong Rong

**Affiliations:** Cixi Center for Disease Control and Prevention, Cixi, China

**Keywords:** adolescents, mediation effect, peer smoking, smoking behavior, smoking intention

## Abstract

**Background:**

Reducing adolescents’ smoking rates is an important priority for global public health. Smoking intention is the most direct factor influencing smoking behavior and the best predictor of its occurrence. Smoking intention is influenced by peer smoking behavior, which strengthens adolescents’ willingness to smoke. Understanding adolescents’ smoking intentions and their influencing factors will be a key component of future tobacco control. This study aims to explore the relationship between peer smoking and adolescents’ future smoking intentions, investigate the potential roles of beliefs and attitudes toward smoking behavior in this process, and identify the pathways among these variables.

**Method:**

A multi-stage stratified cluster sampling method was adopted to select 4,617 students from 16 junior and senior high schools in Cixi City as study subjects. The *2023 China High School Students Tobacco Use Survey Questionnaire* was used to investigate data such as students’ basic demographics, awareness, and usage status of tobacco products. Structural Equation Modeling (SEM) was employed to analyze the impact of peer smoking on adolescents’ future smoking intentions, as well as the mediating roles of beliefs and attitudes toward smoking behavior.

**Results:**

Among participants, 2,359 (51.1%) were male, and 2,258 (48.9%) were female. SEM showed a significant mediating effect of beliefs and attitudes toward smoking behavior in the relationship between peer smoking and adolescents’ future smoking intentions. This mediating effect consists of two pathways: the independent mediating effect of attitudes toward smoking behavior, and the chain mediating effect of beliefs and attitudes toward smoking behavior. Through bootstrap testing, the standardized total effect was 0.233 (0.185–0.280), the standardized direct effect was 0.167 (0.119–0.216), accounting for 71.7% of the standardized total effect, and the standardized indirect effect was 0.066 (0.049–0.081), accounting for 28.3%.

**Conclusion:**

Peer smoking not only directly enhances adolescents’ future smoking intentions but also indirectly reinforces this effect by altering their beliefs and attitudes toward smoking behavior. Therefore, conducting precise interventions targeting beliefs and attitudes toward smoking behavior can effectively mitigate the negative influence of peer smoking, thereby providing empirical evidence for adolescent tobacco control strategies.

## 1 Introduction

Smoking causes over 8 million deaths and 200 million disability-adjusted life years globally each year (1). According to the *Report on the Health Hazards of Smoking in China 2020*, the number of smokers in China exceeds 300 million, with a smoking rate of 26.6% among the population aged 15 and above. Smoking causes over 1 million deaths annually in China, exceeding the total number of deaths from AIDS, tuberculosis, traffic accidents, and suicide combined. If the smoking rate cannot be effectively controlled, this figure is projected to rise to 2 million deaths annually by 2030 and 3 million by 2050.

Smoking behavior is primarily formed during adolescence, and starting to smoke during adolescence leads to higher addiction rates in adulthood (2). Therefore, implementing tobacco control work among adolescents is a critical strategy for reducing adult smoking rates. However, data from the Global School-based Student Health Survey (GSHS) shows that among 23 participating countries, adolescents’ smoking rates rose in 4 countries and showed no difference in 13 countries; smoking rates were higher among males, those exposed to secondhand smoke, and adolescents in upper-middle-income countries (3). A 2022 survey of risk behaviors among adolescents in Zhejiang Province, China, which involved 24,835 high school students, revealed that 998 were current smokers, accounting for 4.02% of the participants (4). Reducing adolescents’ smoking rates remains an important priority for global public health. The Theory of Planned Behavior (TPB) holds that before implementing a certain behavior, individuals must possess the willingness to perform the behavioral trend, namely, behavioral intention, representing the likelihood of an individual’s readiness to perform a certain behavior. Numerous studies have confirmed that smoking intention is the most direct factor influencing smoking behavior and the best predictor of its occurrence, and researchers have reduced tobacco use by lowering future smoking intentions (5–7). Therefore, it is necessary to understand adolescents’ smoking intentions and their influencing factors, which will constitute a key component of future tobacco control.

Previous studies have indicated that peer smoking is the strongest predictor of non-smokers’ smoking intentions, with an influence even exceeding that of parental smoking (8, 9). Peers are commonly important figures around adolescents, and play a crucial role in their social environments. According to Social Learning Theory, an individual’s smoking intentions and behaviors can be acquired through observing others’ smoking behaviors. In this process, observational learning and vicarious reinforcement play key roles (10). This suggests that smoking intention is influenced by the surrounding environment, and peer smoking behaviors may serve as models for observational learning among adolescents, thereby strengthening their willingness to smoke.

Belief is the probability that an individual subjectively considers that adopting a certain behavior will cause a certain result, while attitude is an individual’s general and stable tendency or stance toward a certain behavior. The difference between the two lies in the fact that beliefs focus more on cognitive judgments about the consequences of behavior (e.g., smoking makes me look less attractive). Attitudes, on the other hand, represent an emotional inclination toward the behavior itself (e.g., I dislike smoking). In the process of people obtaining and utilizing information, they gradually form individual knowledge, beliefs, and values, and form attitudes on this basis. Within adolescent groups, those who smoke may impose negative values on other members of the group to align them with the group’s expectations and may alienate friends whose beliefs, attitudes, and values are inconsistent with their own. Non-smoking adolescents may perceive risky behaviors such as smoking as a way to gain peer acceptance, thereby changing their beliefs and attitudes toward smoking behavior (11, 12). According to the Theory of Reasoned Action (TRA) and the Theory of Planned Behavior (TPB), smoking attitudes directly influence smoking intentions. In some behavioral studies, the correlation between attitude and behavioral intention is the strongest, and attitude plays a partial mediating role between belief and behavioral intention (13–15).

Previous studies have predominantly focused on factors associated with smoking intention and have confirmed the effect of peer smoking on adolescents’ smoking intentions. However, there are few studies on the mechanism underlying the influence of peer smoking on smoking intentions and the mediating effects of smoking beliefs and attitudes. To supplement relevant research evidence, implement precise tobacco control strategies among adolescents, and reduce adolescents’ smoking rates, this study, based on the aforementioned theoretical foundations and previous research findings, proposes the following research hypotheses: (1) Peer smoking can directly influence adolescents’ smoking intentions; (2) Attitude toward smoking behavior play a mediating effect in the relationship between peer smoking and smoking intention; and (3) Beliefs and attitudes toward smoking behavior play a chain mediating effect in the relationship between peer smoking and smoking intention. These hypotheses aim to suggest that conducting precise interventions targeting beliefs and attitudes toward smoking behavior can effectively mitigate the negative influence of peer smoking.

## 2 Materials and methods

### 2.1 Study design

The minimum sample size for the adolescent tobacco survey was calculated using the estimation formula: $N=\frac{\mu_{\alpha }^{2}\times p(1-p)}{\delta^{2}}\times \text{deff}$ Based on the current smoking rate of 4.35% among the population aged 15–24 in our city in 2022, *p* = 0.0435; setting the allowable relative error at 15%, the allowable absolute error *δ* is 15% × 4.35%, *μ_α_* = 1.96, and *deff* = 1. The calculated minimum sample size was 3,754 individuals. The survey employed a stratified cluster random sampling method. After stratifying by junior high school, general senior high school, and vocational high school, 8 junior high schools, 5 general senior high schools, and 3 vocational high schools were randomly selected. Then, 2 classes were randomly selected from each grade, with a total of 6 classes surveyed per school. All students present in the selected classes on the survey day constituted the subjects of the study, yielding a total sample of 4,617 individuals.

### 2.2 Survey method

The survey was conducted during lunch break in school computer labs, with the class serving as the unit. Each student opened the questionnaire link via a web browser and completed it independently; discussion was not allowed. One surveyor supervised the entire process. The testing time was approximately 30 min. During the testing process, students could raise their hands to ask if they encountered any questions they did not understand.

### 2.3 Selection of indicators

#### 2.3.1 Basic information

School type, gender, parental smoking, and tobacco advertising exposure. All of the above factors have been established in theory and previous research as key predictors of adolescents’ smoking intention (16, 17), and should be incorporated into the model to control for their potential influence on adolescents’ smoking intention.

#### 2.3.2 Peer smoking

Measured by the question (18), “Do any of your close friends smoke?” Responses were recorded on a 2-point scale: “Yes”(scored 1) and “No” (scored 0).

#### 2.3.3 Beliefs toward smoking behavior

Measured by the question, “Do you think smoking makes young people look more or less attractive?” Responses were recorded on a 3-point scale: “Less attractive” (scored 1), “No difference compared to non-smokers” (scored 2), and “More attractive” (scored 3). A higher score indicates a stronger belief that smoking makes young people more attractive.

#### 2.3.4 Attitudes toward smoking behavior

Measured by the question, “Do you agree or disagree with the following statement: I think I might enjoy smoking cigarettes?” Responses were recorded on a 4-point scale: “Strongly disagree” (scored 1), “Disagree” (scored 2), “Agree” (scored 3), and “Strongly agree” (scored 4). A higher score indicates a higher likelihood of having an attitude that one would enjoy smoking cigarettes.

#### 2.3.5 Future smoking intentions

Measured by the question, “Do you think you will use any tobacco product in the next 12 months?” Responses were recorded on a 4-point scale: “Definitely not” (scored 1), “Probably not” (scored 2), “Probably yes” (scored 3), and “Definitely yes” (scored 4). A higher score indicates a higher likelihood that one will use tobacco products in the next 12 months.

### 2.4 Quality control

The researchers underwent rigorous training and were proficient in the questionnaire filing rules. During data cleaning, a questionnaire was deemed invalid if the proportion of unanswered items in a questionnaire exceeded 20% or if logical inconsistencies were identified in the answers. A total of 4,655 questionnaires were collected. After excluding 38 invalid questionnaires, the remaining 4,617 valid questionnaires were used for data analysis.

### 2.5 Statistical methods

Data were exported from the Questionnaire Star. Statistical description and analysis were performed using SPSS 25.0. Qualitative data were described using frequencies and percentages. Rank correlation analysis was employed to analyze the correlations among school type, gender, parental smoking, tobacco advertising, peer smoking, beliefs toward smoking behavior, attitudes toward smoking behavior, and smoking intention for the next 12 months. The study features a large sample size, and the correlation coefficients among the predictor variables are moderate, indicating that multicollinearity is not an issue. Mediating effects between dependent and independent variables were tested using the R 4.3.1 “lavaan” package, with the “WLSMV” estimation method and 5,000 Bootstrap samples. The Comparative Fit Index (CFI), Tucker-Lewis Index (TLI), Root Mean Square Error of Approximation (RMSEA), and Standardized Root Mean Square Residual (SRMR) were used to assess model fit. CFI and TLI values above 0.90 indicate acceptable fit, while values above 0.95 denote good fit. RMSEA values below 0.08 indicate acceptable fit, while values below 0.05 indicate good fit; SRMR values below 0.08 indicate good model fit. The statistical significance level was set at *α* = 0.05.

## 3 Results

### 3.1 Descriptive statistics

A total of 4,617 people were surveyed. Among them, 2,359 (51.1%) were male and 2,258 (48.9%) were female; 2,212 students (47.9%) were from junior high schools, 1,984 students (43.0%) had at least one parent who smoked, 1,108 students (24.0%) reported exposure to tobacco advertising, and 592 students (12.8%) had peers who smoked (Table 1).

**Table 1:** Basic information (*n* = 4,617).

Variables	Number of cases	Proportion (%)*
School type		
Junior high schools	2,212	47.9
General senior high schools	1,530	33.1
Vocational senior high schools	875	19.0
Gender		
Male	2,359	51.1
Female	2,258	48.9
Parental smoking		
At least one parent	1984	43.0
Neither	2,633	57.0
Tobacco advertising exposure		
Yes	1,108	24.0
No	3,509	76.0
Peer smoking		
Yes	592	12.8
No	4,025	87.2
Beliefs toward smoking behavior		
Decreases attractiveness	2,957	64.0
No difference in attractiveness	1,326	28.7
Increases attractiveness	334	7.2
Attitudes toward smoking behavior		
Strongly disagreed	3,397	73.6
Disagreed	925	20.0
Agreed	114	2.5
Strongly agreed	181	3.9
Future smoking intention in the next 12 months		
Definitely would not use	4,407	95.5
Probably not	115	2.5
Probably yes	56	1.2
Definitely yes	39	0.8

*Indicates that the sum of percentages may not equal 100% due to rounding.

Regarding beliefs toward smoking behavior: 334 students (7.2%) thought that smoking increases the attractiveness of young people, 1,326 students (28.7%) perceived no difference in attractiveness, and 2,957 students (64.0%) thought that smoking decreases attractiveness. Regarding attitudes toward smoking behavior: for the statement “I might enjoy smoking cigarettes, 3,397 students (73.6%) strongly disagreed, 925 students (20.0%) disagreed, 114 students (2.5%) agreed, and 181 students (3.9%) strongly agreed. Regarding future smoking intention in the next 12 months: 4,407 students (95.5%) thought they would definitely not use any tobacco product, 115 students (2.5%) thought probably not, 56 students (1.2%) thought probably yes, and 39 students (0.8%) thought definitely yes.

### 3.2 Correlation analysis

Correlation analysis results (Table 2) showed that school type was correlated with peer smoking (*r* = 0.098, *p* < 0.001). Gender was correlated with peer smoking (*r* = −0.145, *p* < 0.001). Compared to males, females scored lower on beliefs toward smoking behavior (*r* = −0.182, *p* < 0.001), attitudes toward smoking behavior (*r* = −0.117, *p* < 0.001), and future smoking intention in the next 12 months (*r* = −0.084, *p* < 0.001). Parental smoking was positively correlated with peer smoking (*r* = 0.159, *p* < 0.001). Compared to adolescents with non-smoking parents, those with at least one smoking parent scored higher on future smoking intention in the next 12 months (*r* = 0.030, *p* < 0.001). Tobacco advertising exposure was positively correlated with peer smoking (*r* = 0.146, *p* < 0.001). Compared to those not exposed to tobacco advertising, individuals exposed to tobacco advertising scored higher on attitudes toward smoking behavior (*r* = 0.100, *p* < 0.001) and future smoking intention in the next 12 months (*r* = 0.081, *p* < 0.001). Beliefs toward smoking behavior were positively correlated with attitudes toward smoking behavior (*r* = 0.184, *p* < 0.001) and future smoking intention in the next 12 months (*r* = 0.111, *p* < 0.001). Attitudes toward smoking behavior were positively correlated with future smoking intention in the next 12 months (*r* = 0.200, *p* < 0.001). Compared to those with non-smoking peers, individuals with smoking peers scored higher on beliefs toward smoking behavior (*r* = 0.072, *p* < 0.001), attitudes toward smoking behavior (*r* = 0.174, *p* < 0.001), and future smoking intention in the next 12 months (*r* = 0.190, *p* < 0.001).

**Table 2:** Correlation between variables (*n* = 4,617).

Variables	School type	Gender	Parental smoking	Tobacco advertising exposure	Peer smoking	Beliefs toward smoking behavior	Attitudes toward smoking behavior	Future smoking intention in the next 12 months
School type	1							
Gender	0.014	1						
Parental smoking	0.070*	−0.006	1					
Tobacco advertising exposure	0.046*	−0.046*	0.142*	1				
Peer smoking	0.098*	−0.145*	0.159*	0.146*	1			
Beliefs toward smoking behavior	0.025	−0.182*	−0.017	0.028	0.072*	1		
Attitudes toward smoking behavior	0.024	−0.117*	0.024	0.100*	0.174*	0.184*	1	
Future smoking intention in the next 12 months	−0.002	−0.084*	0.030*	0.081*	0.190*	0.111*	0.200*	1

*Represents *p* < 0.001.

### 3.3 Mediating effect analysis

Separate regression models were constructed with beliefs toward smoking behavior, attitudes toward smoking behavior, and future smoking intention in the next 12 months as dependent variables, and peer smoking as an independent variable, while controlling for school type, gender, parental smoking, and tobacco advertising exposure. The results are shown in Table 3.

**Table 3:** Regression analysis results of the chain mediation effect model.

Variables	Model 1: beliefs toward smoking behavior				Model 2: attitudes toward smoking behavior				Model 3: future smoking intention in the next 12 months			
	*β*	SE	*z*	*p*	*β*	SE	*z*	*p*	*β*	SE	*z*	*p*
School type												
General senior high schools	−0.055	0.041	−1.331	0.183	0.086	0.046	1.870	0.061	−0.140	0.084	−1.669	0.095
Vocational senior high schools	0.095	0.050	1.908	0.056	−0.069	0.054	−1.286	0.198	−0.085	0.093	−0.911	0.363
Gender	−0.425	0.037	−11.566	< 0.001	−0.145	0.042	−3.440	0.001	−0.219	0.077	−2.825	0.005
Parental smoking	−0.094	0.038	−2.465	0.014	−0.017	0.042	−0.394	0.694	−0.028	0.075	−0.375	0.708
Tobacco advertising exposure	0.055	0.042	1.313	0.189	0.225	0.046	4.850	<0.001	0.154	0.080	1.939	0.052
Peer smoking	0.184	0.053	3.488	<0.001	0.530	0.055	9.665	<0.001	0.567	0.086	6.629	<0.001
Beliefs toward smoking behavior					0.327	0.023	14.182	<0.001				
Attitudes toward smoking behavior									0.374	0.037	10.058	<0.001
*R* ^2^	0.056				0.160				0.222			

Model 1 took beliefs toward smoking behavior as the dependent variable, gender (*β* = −0.425, *p* < 0.001), and parental smoking (*β* = −0.094, *p* < 0.001) were correlated with beliefs toward smoking behavior. Peer smoking could positively influence adolescents’ beliefs toward smoking behavior (*β* = 0.184, *p* < 0.001).

Model 2 took attitudes toward smoking behavior as the dependent variable, adding beliefs toward smoking behavior as an independent variable on the basis of Model 1. The results showed that peer smoking (*β* = 0.530, *p* < 0.001) and beliefs toward smoking behavior (*β* = 0.327, *p* < 0.001) were positively correlated with attitudes toward smoking behavior, and gender (*β* = −0.145, *p* = 0.001) and tobacco advertising exposure (*β* = 0.225, *p* < 0.001) were both correlated with attitudes toward smoking behavior.

Model 3 took future smoking intention in the next 12 months as the dependent variable, further adding attitudes toward smoking behavior as an independent variable on the basis of Model 2. The results showed that peer smoking (*β* = 0.567, *p* < 0.001) and attitudes toward smoking behavior (*β* = 0.374, *p* < 0.001) were positively correlated with future smoking intention in the next 12 months. Gender (*β* = −0.219, *p* = 0.005) was correlated with future smoking intention in the next 12 months.

**Figure 1:**
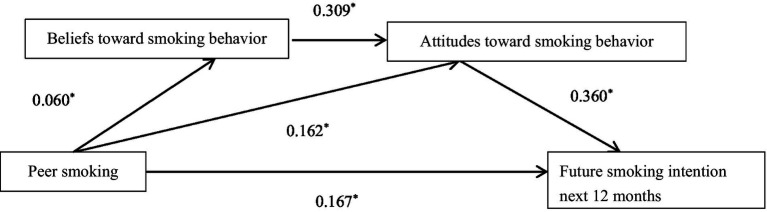
Chain mediation effect model. *Represents *p* < 0.001. Path coefficients were standardized.

A chain mediation model was constructed to further explore the potential mediating effects of beliefs and attitudes toward smoking behavior on future smoking intention in the next 12 months. When the model pathways were specified as shown in Figure 1, the measurement model demonstrated a good fit with the data: CFI = 0.976, TLI = 0.927, RMSEA = 0.044, SRMR = 0.031. There are two pathways for the influence of peer smoking on adolescents’ future smoking intention. Pathway 1: Peer smoking→Attitudes toward smoking behavior→Future smoking intention in the next 12 months; Pathway 2: Peer smoking→Beliefs toward smoking behavior→Attitudes toward smoking behavior→Future smoking intention in the next 12 months. Through bootstrap testing, the standardized total effect was 0.233 (0.185–0.280), the standardized direct effect was 0.167 (0.119–0.216), accounting for 71.7% of the standardized total effect, and the standardized indirect effect was 0.066 (0.049–0.081), accounting for 28.3% (Table 4).

**Table 4:** Bootstrap testing results of the standardized total effect, direct effect, and indirect effect of the model.

Mechanism	Effect	*p*	95% CI	Contribution rate (%)
Total effect	0.233	<0.001	0.185–0.280	100.0
Direct effect	0.167	<0.001	0.119–0.216	71.7
Indirect effect	0.066	<0.001	0.049–0.081	28.3
Pathway 1	0.059	<0.001	0.043–0.074	25.3
Pathway 2	0.007	0.001	0.003–0.011	3.0

Pathway 1: Peer smoking→Attitudes toward smoking behavior→Future smoking intention in the next 12 months. Pathway 2: Peer smoking→Beliefs toward smoking behavior→Attitudes toward smoking behavior→Future smoking intention in the next 12 months.

## 4 Discussion

This study surveyed a total of 4,617 students from 16 junior and senior high schools. Among the subjects, 12.8% reported having peers who smoked, a proportion lower than the survey data of adolescents in five European countries (19). The study focused on exploring the influence of peer smoking on Chinese adolescents’ smoking intentions, constructed a chain mediation model involving peer smoking, beliefs toward smoking behavior, and attitudes toward smoking behavior, and tested its validity in accordance with the study objectives.

### 4.1 Direct effect of peer smoking on adolescents’ smoking intentions

This study found that peer smoking can directly and positively influence adolescents’ smoking intention, and the direct effect accounts for the majority (72.0%) of the total effect. This result is consistent with previous studies (20, 21). This result further validates the critical role of peer smoking in reinforcing adolescents’ smoking intentions. According to Social Learning Theory, the development of individual behavior is influenced not only by personal experience but also by the behaviors of others and environmental factors; that is, individuals can change their own behavioral patterns by observing the behaviors and consequent outcomes experienced by others (22). Adolescents can form behavioral intentions directly through observation and imitation of the behaviors of their peers (particularly those who are popular or admired), without necessarily undergoing complex cognitive processes (23). Smoking may function as a “pass” to enter into a certain social circle, and direct imitation serves as a strong signal of seeking acceptance and belonging (24). This represents a rapid, quasi-instinctual response driven by motivations such as “looking cool” or “wanting to fit in. Perceived social norms regarding smoking refer to an individual’s perception of others’ attitudes toward smoking and their actual smoking behaviors, which are considered predictors of adolescent smoking (25). The Theory of Planned Behavior posits that perceived smoking social norms are a key environmental factor inducing smoking behavior, which influences behavior through their effect on behavioral intention. This theory has been supported by a large number of empirical studies (26, 27). Peer behavior directly shapes adolescents’ judgment of “normal behavior. If they see many peers smoking, they will think “everyone does it, which is a powerful social norm pressure that directly drives intention, bypassing personal beliefs and attitudes.

### 4.2 Attitudes toward smoking behavior play a partial mediating role between peer smoking and smoking intention

This study found that attitudes toward smoking behavior played a partial mediating role, accounting for 25.3% of the total effect. This indicates that peer smoking not only directly leads to smoking intention but may also indirectly influence it by altering adolescents’ attitudes towards smoking itself. The Health Belief Model Theory holds that perceptions play a decisive role in the formation and maintenance of health behaviors (28). Adolescents see many peers smoking without suffering immediate negative consequences, which lowers their perception of smoking risks, making their attitudes less negative. Furthermore, seeing peers showing enjoyment, relaxation, more confidence, or a cooler appearance while smoking, non-smoking adolescents may associate these positive emotional experiences with smoking behavior, thereby forming more positive attitudes towards smoking (29). According to the Theory of Planned Behavior, positive smoking attitudes directly influence smoking intention.

### 4.3 Beliefs and attitudes toward smoking behavior play a chain mediating role between peer smoking and smoking intention

This study further revealed a complete pathway of peer smoking influence from cognition to affect and then to behavioral intention. Social Cognitive Theory emphasizes the role of personal cognitive factors. During cognitive processing, the brain encodes and stores specific information, gradually forming an individual’s knowledge, and on this basis forms attitudes which then influence behavioral intentions (30). In this study, peer smoking may first influenced beliefs about smoking behavior. Through interactions with smoking peers, adolescents may be exposed to misinformation about smoking, such as “smoking can relieve stress”, “facilitates socialization”, or “smoking is cool”, etc., leading non-smoking adolescents to develop more positive perceptions and evaluations of smoking behavior. According to the Theory of Reasoned Action Model, attitudes are determined jointly by beliefs and the evaluation of the belief outcomes. If the outcomes of these peer-reinforced smoking beliefs are evaluated as positive by the individual, a general positive attitude will be formed (25, 31). Ultimately, this formed positive attitude predicts behavioral intention.

## 5 Limitations

This study employed a simplified approach to assess beliefs toward smoking behavior, measuring only by the question “Do you think smoking makes young people look more or less attractive?” This limited measurement may have underestimated the broader influence of peer smoking on beliefs toward smoking behavior. This study is a cross-sectional survey, and the mediation effect model reflects a theoretically consistent path rather than a causal relationship. In the future, longitudinal studies can be conducted to validate the results of this study. Self-reported data collected through student questionnaires may be subject to reporting bias, leading to discrepancies between the obtained data and actual circumstances.

## 6 Conclusion

This study constructed and validated a chain mediation model, which not only confirms the existence of peer smoking’s influence on adolescents’ smoking intention but also delineates a clear psychological roadmap of how it functions, deepening and refining the understanding of the mechanism of peer smoking influence. The influence of peer smoking on adolescents operates through two distinct yet concurrent pathways: one is a “fast track” — powerful, direct, based on imitation and normative pressure. The other is a “slow track” — more refined, based on cognition, functioning by changing beliefs and attitudes. The discovery of this chain mediation holds crucial implications for designing precise intervention measures. If one wants to intervene by changing cognition, merely publicizing that “smoking is harmful” may be too general. Instead, interventions should precisely target the specific positive beliefs and attitudes reinforced by peers, for example, by revealing through role-playing that smoking is not truly cool, or by providing healthier ways of socializing.

## Data Availability

The raw data supporting the conclusions of this article will be made available by the authors, without undue reservation.
